# *Talaromyces marneffei* promotes M2-like polarization of human macrophages by downregulating SOCS3 expression and activating the TLR9 pathway

**DOI:** 10.1080/21505594.2021.1958470

**Published:** 2021-08-02

**Authors:** Wudi Wei, Chuanyi Ning, Jiegang Huang, Gang Wang, Jingzhen Lai, Jing Han, Jinhao He, Hong Zhang, Bingyu Liang, Yanyan Liao, Thuy Le, Qiang Luo, Zhen Li, Junjun Jiang, Li Ye, Hao Liang

**Affiliations:** a Guangxi Key Laboratory of AIDS Prevention and Treatment, School of Public Health, Guangxi Medical University, Nanning, Guangxi, China; b Guangxi-ASEAN Collaborative Innovation Center for Major Disease Prevention and Treatment, Life Sciences Institute, Guangxi Medical University, Nanning, Guangxi, China; c Nursing College, Guangxi Medical University, Nanning, Guangxi, China; d Oxford University Clinical Research Unit, Wellcome Trust Major Overseas Programme, Ho Chi Minh City, Vietnam; e Division of Infectious Diseases and International Health, Duke University, Durham, North Carolina, USA

**Keywords:** *Talaromyces marneffei*, macrophages, M2 polarization, SOCS3, TLR9 pathway

## Abstract

Little is known about how *Talaromyces marneffei*, a thermally dimorphic fungus that causes substantial morbidity and mortality in Southeast Asia, evades the human immune system. Polarization of macrophages into fungal-inhibiting M1-like and fungal-promoting M2-like types has been shown to play an important role in the innate immune response against fungal pathogens. This mechanism has not been defined for *T. marneffei*. Here, we demonstrated that *T. marneffei* promotes its survival in human macrophages by inducing them toward M2-like polarization. Our investigations of the mechanism revealed that *T. marneffei* infection led to SOCS3 protein degradation by inducing tyrosine phosphorylation, thereby relieving the inhibitory effect of SOCS3 on p-STAT6, a key factor for M2-like polarization. Our SOCS3-overexpression experiments showed that SOCS3 is a positive regulator of M1-like polarization and plays an important role in limiting M2-like polarization. Furthermore, we found that inhibition of the TLR9 pathway partially blocked *T. marneffei*-induced M2-like polarization and significantly enhanced the killing activity of macrophages against *T. marneffei*. Collectively, these results reveal a novel mechanism by which *T. marneffei* evades the immune response of human macrophages.

## Introduction

*Talaromyces marneffei* (formerly *Penicillium marneffei*) is a thermally dimorphic fungus endemic in southeast Asia, southern China, and northeastern India and causes an invasive fungal infection in immunocompromised individuals living in or traveling to these endemic regions[1], [2]. Human infection is presumed to occur in the lung via inhalation of aerosolized conidia from the environment which, in the setting of immunosuppression, can cause a life-threatening multi-organ disseminated infection through the reticuloendothelial system [2]. Advanced HIV disease (CD4 cell count <100 cells/mm [3]) is a major risk factor for *talaromycosis*. In the highly endemic regions of northern Thailand, Vietnam, and southern China, *talaromycosis* is diagnosed in up to 16% of HIV hospital admissions, and is a leading cause of HIV-associated death [3,4]. Infections are increasingly diagnosed in non-HIV-infected people who have a primary immunodeficiency condition (such as idiopathic CD4 lymphopenia, anti-interferon-gamma autoantibodies, mutations in the CYBB, CD40L, or STAT pathways) and in those who have a secondary immunodeficiency condition (corticosteroids or immunosuppressive therapy, malignancies, and solid or bone marrow transplantation) [5]. The mortality despite antifungal therapy is up to 30% in HIV-infected individuals and up to 50% non-HIV-infected individuals [6–8].

Despite high morbidity and mortality and a growing number of susceptible individuals, our current knowledge of the pathogenicity of *T. marneffei* causing human disease is limited, especially in the dissemination phase. The strategy in which *T. marneffei* evades the immune response during the dissemination phase is unknown. Specialized phagocytes of the immune system provide the important line of defense against fungal infection [9], among them, the activation of macrophages is critical for their anti-fungal function. Macrophages are customarily activated into two functionally distinct M1-like and M2-like phenotypes ^10^. M1-like macrophages are associated with an immune response to intracellular pathogens and are involved in pro-inflammatory responses governed by Toll-like receptors (TLRs) and Th1 interferon gamma signaling. M2-like macrophages are associated with an immune response to helminth infections and asthma and allergies and are involved in anti-inflammatory responses and tissue repair governed by Th2 signaling [10]. *T. marneffei* infection has been shown to induce M2-type factor, IL-10, in mouse alveolar macrophage model and human macrophage model [11,12]. A recent study using zebrafish model showed that macrophages protect *T. marneffei* conidia from neutrophil antifungal activity; however, the specific mechanism is unknown [13]. Since the strategy for immune evasion by promoting macrophage M2-like polarization has been proposed for other intracellular pathogens including *Candida albicans* and *Staphylococcus aureus* [14–16], we hypothesized that *T. marneffei* may evade macrophage antimicrobial functions similarly by inducing M2-like polarization.

Previous studies have identified several proteins and pathways that regulate macrophage polarization, including the suppressor of cytokine signaling (SOCS) family and the JAK-STAT pathway [10,17,18]. Among the SOCS family, SOCS3 has been shown to regulate M1-like polarization in macrophage [19], whereas the JAK-STAT6 pathway is involved in the M2-like polarization [18]. Upon activation by IL-4/IL-13, STAT6 is phosphorylated and then enters the nucleus, leading to the production of M2-related factors; this process is shown to be regulated by SOCS3 [20–23]. In addition, several pattern recognition receptors (PRRs) like Dectin-1, 2, 3, and TLR4, 9, are expressed in macrophages and are involved in fungal cell or DNA recognition [24–26]. However, the role of SOCS3 and TLR9 play in the promotion of macrophage polarization in *T. marneffei* infection has not been defined.

In this study, we showed that *T. marneffei* evades the antifungal activity of human macrophages by inducing them toward M2-like polarization, rendering them permissive for fungal proliferation. We showed that SOCS3 is a positive regulator of M1-like polarization in human macrophages, and we showed that *T. marneffei* promotes macrophage M2-like polarization by directly down regulating SOCS3 and activating the TLR9 pathway.

## Results

### *The antifungal response of macrophages against* T. marneffei *infection is associated with macrophage polarization status.*

**Figure 1.**
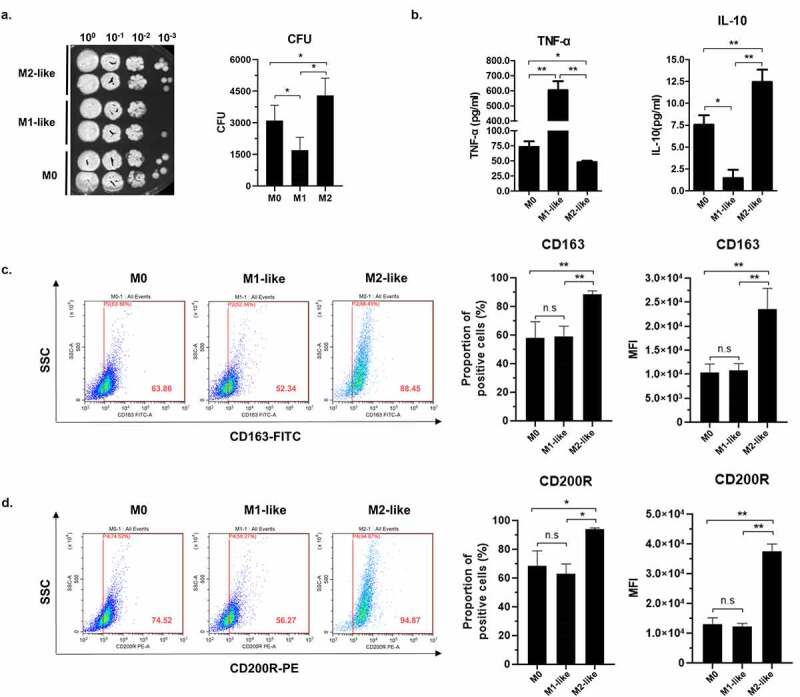
Association between human THP-1 macrophage phenotypes and antifungal response to *T. marneffei*. PMA-treated (M0), PMA/LPS/IFN-γ-treated (M1-like) or PMA/IL-4-treated (M2-like) THP-1 macrophages were incubated with *T. marneffei* spores (MOI = 10) for 24 h, respectively. (a) *T. marneffei* colony forming units (CFU) by microdilution spot assay to assess antifungal activity of M0, M1-like, M2-like macrophages. The culture supernatants and cell lysates were collected, harvested *T. marneffei* by centrifugation. CFUs microdilution spot assay was conducted to measured antifungal ability of M0, M1-like or M2-like macrophages. (b) Levels of TNF-α and IL-10 in M0, M1-like or M2-like macrophages as measured by the cytometric bead array system (CBA). (c and d) Levels of CD163 (c) and CD200R (d) in M0, M1-like or M2-like macrophages as measured by the flow cytometry. All data were shown as mean ± SD of the results of at least three independent experiments (*, *p* < 0.05, **, *p* < 0.01, n.s, no significant difference, by Student’s t-test)

It is now known that M1-like macrophages have high secretion of pro-inflammatory cytokines such as TNF-α and IL-1β, and play an important role during immune responses against pathogens, whereas M2-like macrophages dampen their antifungal response by the increased expression of CD163 and CD200R, the most commonly used M2 biomarker, and the secretion of IL-10 [18,27]. In order to investigate human THP1 macrophages polarization status in response to *T. marneffei*, we challenged the PMA-treated macrophages (M0), PMA/LPS/IFN-γ-treated macrophages (M1-like), and PMA/IL-4-treated macrophages (M2-like) with *T. marneffei* spores, respectively. As shown in Figure 1a, The *T. marneffei* colony forming units (CFUs) in microdilution spot assay showed that M1-like macrophages had the strongest killing activity against *T. marneffei*, whereas M2-like macrophages had the weakest killing activity, which shows that M2-like polarization is beneficial to TM survival. To confirm whether different treatments of macrophages induced the corresponding polarization status, we measured the levels of a prototypical cytokine produced by M1-like macrophages, TNF-α, and a cytokine produced by M2-like macrophages, IL-10, in three kinds of macrophages, respectively. We showed that M1-like macrophages had the highest level of TNF-α and the lowest level of IL-10, while M2-like macrophages had the lowest level of TNF-α and the highest level of IL-10 (Figure 1b). Also, flow cytometry showed that M2-like macrophages had the highest level of CD163 and CD200R. These findings are consistent with the characteristics of polarized status of M1-like macrophages or M2-like macrophages.

### T. marneffei *infection induces human THP-1 macrophages toward M2-like polarization.*

**Figure 2.**
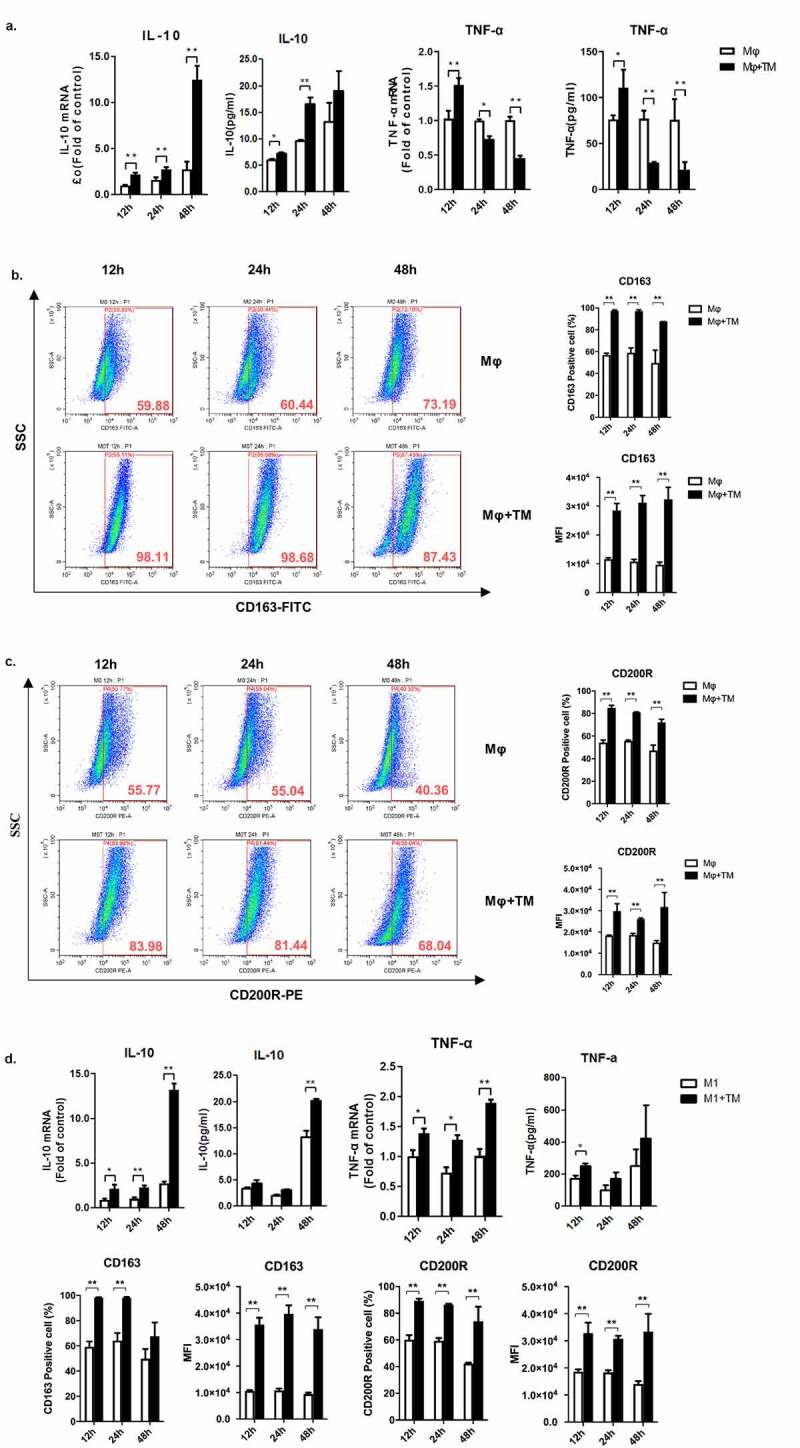
*T. marneffei* infection promotes M2-like polarization of human THP-1 macrophage. The human THP-1 macrophages (PMA-treated) or M1-like macrophages (PMA/LPS/IFN-γ-treated) were infected with *T. marneffei* spores (MOI = 10) for 12 h, 24 h, or 48 h. (a) TNF-α and IL-10 production in *T. marneffei*-infected THP-1 macrophages at 12 h, 24 h, 48 h post-infection, as measured by qRT-PCR and the cytometric bead array system (CBA). (b, c) Levels of CD163 (b) and CD200R (c) in *T. marneffei*-infected macrophages at 12 h, 24 h, 48 h post-infection as measured by flow cytometry. (d) Levels of TNF-α and IL-10 (measured by qRT-PCR and CBA) and the expression of CD163 and CD200R (measured by flow cytometry) in *T. marneffei*-infected M1-like macrophages at 12 h, 24 h, 48 h post-infection. All the levels of mRNA expression were normalized to GAPDH and compared with 12 h timepoint controls. All data were shown as mean ± SD of the results of at least three independent experiments (*, *p* < 0.05, **, *p* < 0.01, by Student’s t-test)

To investigate the impact of *T. marneffei* infection on macrophage polarization, THP-1 macrophages (M0) were infected with *T. marneffei* spores, and the levels of TNF-α and IL-10 were determined by qRT-PCR or cytometric bead array system (CBA). Figure 2a showed that *T. marneffei* infection acted in a time-dependent manner to promote the production of IL-10. Although TNF-α was upregulated at 12 h post-infection, it was significantly down regulated at 48 h post-infection (Figure 2a). We also investigated whether the expression of M2 macrophage markers, CD163 and CD200R, was affected by *T. marneffei* infection of THP-1 macrophages. Flow cytometry results showed that both CD163 and CD200R were significantly upregulated at 12 h, 24 h, and 48 h post-infection (Figure 2b, c).

Next, we determined whether *T. marneffei* infection reverses the polarization of M1-like macrophages by challenging M1-like macrophages with *T. marneffei* spores. We showed that *T. marneffei* infection significantly upregulated the level of IL-10 at both mRNA and protein levels at 12 h, 24 h, and 48 h post-infection (Figure 2d). Although the mRNA level of TNF-α was also upregulated by *T. marneffei* infection, the expression of TNF-α at the protein level was not significantly challenged by *T. marneffei* infection (Figure 2d). These data demonstrated that *T. marneffei* infection induces M2-like conversion in human THP-1 M1-like macrophages.

### T. marneffei *infection induces human peripheral blood monocytes/macrophages toward M2-like polarization.*

**Figure 3.**
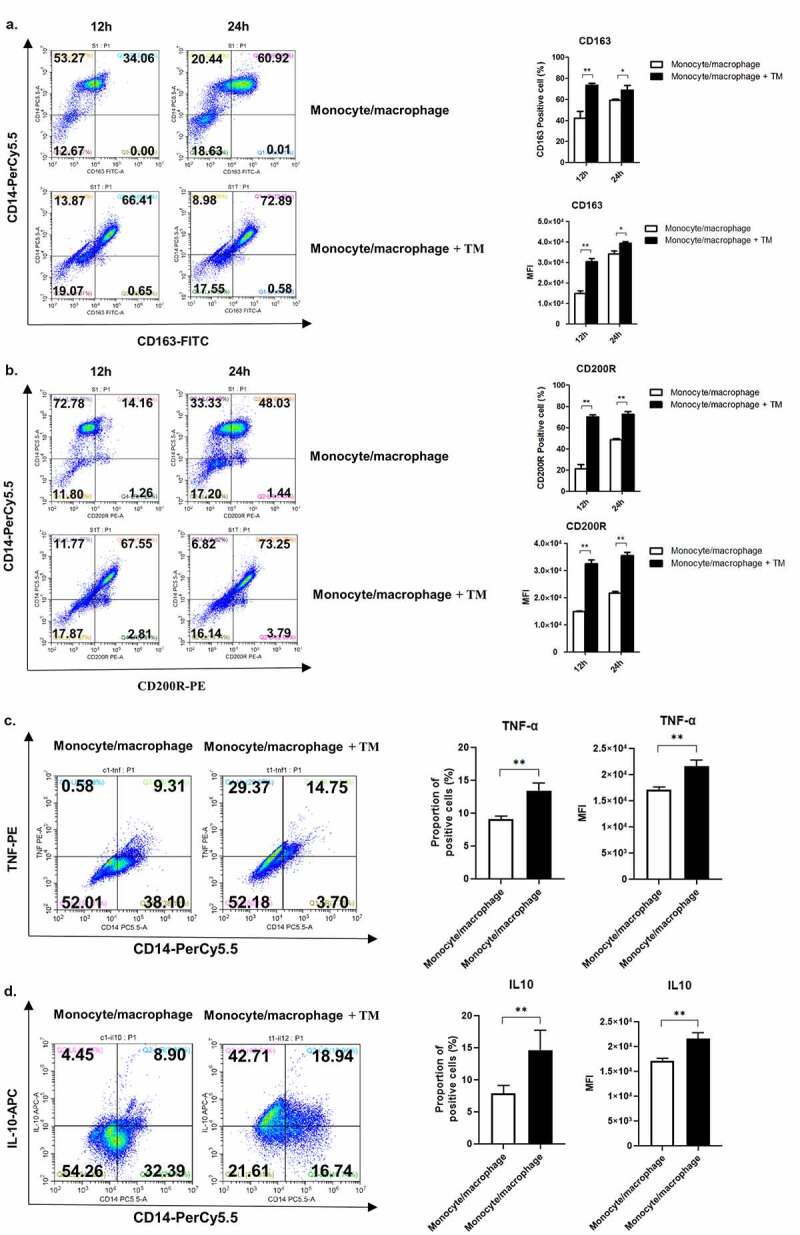
*T. marneffei* infection promotes M2-like polarization of human peripheral blood monocytes. Expression of CD163 (a), CD200R (b), TNF-α (c) and IL-10 (d) in *T. marneffei*-infected peripheral blood monocytes (PBMC). PBMCs were isolated from peripheral blood of healthy people (*n* = 3), then infected with *T. marneffei* spores at a multiplication of infection (MOI) of 10 for 12 h or 24 h. Human PBMCs were marked by CD14. The expression of CD163 and CD200R was determined by flow cytometry. For cytokine detection, the PBMCs were first infected with *T. marneffei* spores as mentioned above for 24 h. And cells were treated with 5 μM of Brefeldin A (BFA) for 6 h before the detection. The data were showed as mean ± SD of results (*, *p* < 0.05, **, *p* < 0.01, by Student’s t-test)

We tested whether *T. marneffei* infection also affects polarization of human peripheral blood monocytes/macrophages. Human peripheral blood mononuclear cells (PBMCs) isolated from healthy subjects were co-cultured with *T. marneffei* spores, and the expression of CD163, CD200R, TNF-α, and IL-10 in peripheral blood monocytes/macrophages (marked by CD14) were measured by flow cytometry. We found that *T. marneffei* infection induced CD163 and CD200R expression in human monocytes/macrophages at 12 h and 24 h post-infection (Figure 3a, b), the same as TNF-α and IL-10 up-regulation at 24 h post-infection (Figure 3c, d), suggesting that *T. marneffei* infection also enhanced polarization toward the M2-like phenotype in human monocytes/macrophages.

### T. marneffei *affects the SOCS3-STAT6 pathway in human macrophages.*

**Figure 4.**
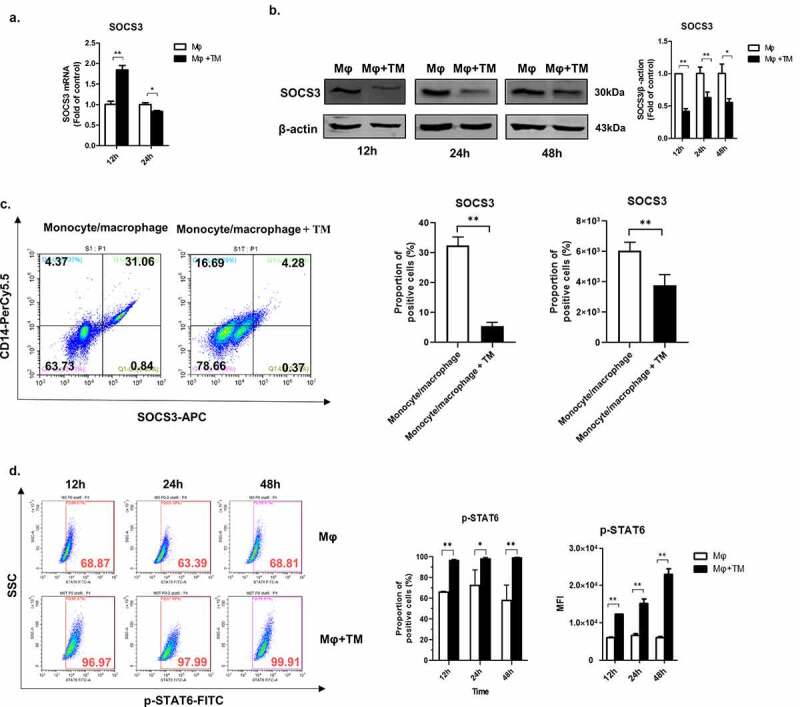
*T. marneffei* infection upregulates SOCS3 and downregulates STAT6 in human macrophages. The human THP-1 macrophages (PMA-treated) were infected with *T. marneffei* spores (MOI = 10) for 24 h. (a) Levels of SOCS3 mRNA in *T. marneffei*-infected THP-1 macrophages at 12 h and 24 h post-infection as measured by qRT-PCR. Levels of mRNA expression were normalized to GAPDH and compared with 12 h timepoint controls. (b) Levels of SOCS3 protein in *T. marneffei*-infected THP-1 macrophages at 12 h, 24 h, 48 h post-infection as measured by Western blot. SOCS3 expression was normalized to ACTIN and compared with 12 h timepoint controls. (c) Levels of p-STAT6 in *T. marneffei*-infected macrophages at 12 h, 24 h, 48 h post-infection as measured by flow cytometry. All data were showed as mean ± SD of results of at least three independent experiments (*, *p* < 0.05, **, *p* < 0.01, by Student’s t-test)

It is well known that the SOCS3-STAT6 pathway plays an important role in controlling macrophage polarization [23,28,29]. In order to investigate the effect of *T. marneffei* infection on the SOCS3-STAT6 pathway, we infected THP-1 macrophages (M0) with *T. marneffei* spores and measured the levels of SOCS3 mRNA and protein. We found that *T. marneffei* infection upregulated SOCS3 mRNA expression at 12 h post-infection; however, a down-regulated effect was observed at 24 h post-infection (Figure 4a). At the protein level, SOCS3 was significantly decreased by *T. marneffei* infection at 12 h, 24 h, 48 h post-infection (Figure 4b). Although there is no obvious time-dependent effect at different time points, *T. marneffei*-infected cells expressed significantly lower level of SOCS3 compared with control cells at different time points (Figure 4b). Similar phenomenon was observed in *T. marneffei*-infected human monocytes/macrophages at 24 h post-infection (Figure 4c). Since SOCS3 has inhibitory impact on the phosphorylation of STAT6, we also measured the levels of p-STAT6 in *T. marneffei*-infected cells and control cells. Consistent with the inhibitory effect of *T. marneffei* infection on SOCS3 expression, *T. marneffei* infection led to significantly increased levels of p-STAT6 (Figure 4d). Therefore, we showed that the SOCS3-STAT6 pathway was significantly affected during *T. marneffei* infection of human macrophages.

### SOCS3 plays a critical role in the polarization in THP-1 macrophages

**Figure 5.**
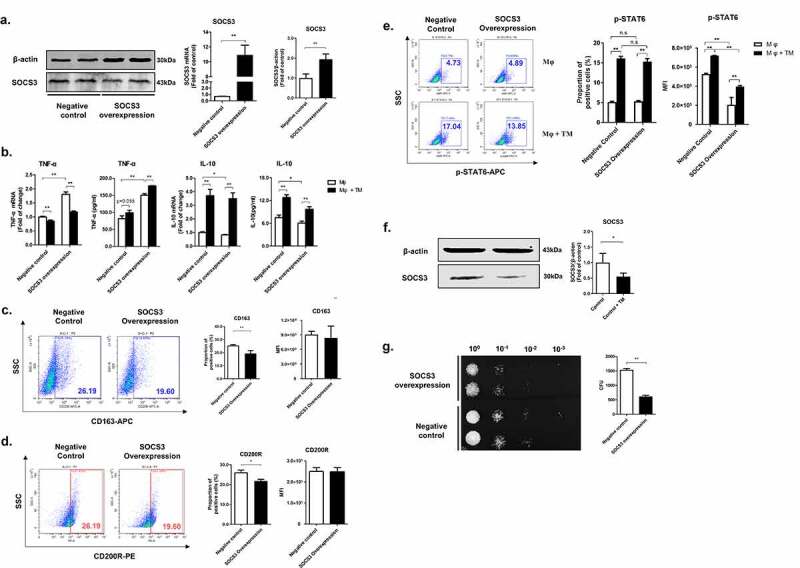
SOCS3 plays an important role in M1-like polarization of human THP-1 macrophages in *T. marneffei* infection. (a) Construction of a SOCS3-overexpressed THP-1 macrophage cell line using lentivirus vector with confirmation by qRT-PCR and Western Blot. (b) Levels of TNF-α and IL-10 in SOCS3- overexpressed THP-1 macrophages and control cells infected or uninfected *T. marneffei* as measured by qRT-PCR and CBA. (c and d) Expression of CD163 (c) and CD200R (d) in SOCS3-overexpressed THP-1 macrophages and control cells as measured by flow cytometry. (e) Levels of p-STAT6 in SOCS3- overexpressed THP-1 macrophages and control cells infected or uninfected *T. marneffei* as measured by flow cytometry. (f) Effects of *T. marneffei* infection on the expression of SOCS3 in SOCS3-overexpressed THP-1 macrophages. (g) Antifungal activity of SOCS3-overexpressed macrophages as measured by CFUs using microdilution spot assay. Levels of mRNA or protein expression were normalized to GAPDH or ACTIN, respectively, and compared with controls. All data were showed as mean ± SD of results of at least three independent experiments (*, *p* < 0.05, **, *p* < 0.01, n.s, no significant difference, by Student’s t-test)

Previous studies have explored the role of SOCS3 in regulating macrophage M1 polarization in mammalian macrophages [17–19]. However, the role of SOCS3 in the polarization of human macrophages would require confirmation through further investigations. To address this question, a SOCS3-overexpressed THP-1 macrophage cell line was constructed using lentivirus vector, which had twice the level of expression of SOCS3 protein compared to the control cells (Figure 5a). The levels of TNF-α and IL-10 in these cells were measured. We showed that SOCS3 overexpression led to significant upregulation of TNF-α at both mRNA and protein level; whereas there was a downregulation of IL-10 expression by SOCS3 overexpression (Figure 5b). Furthermore, SOCS3 overexpression inhibited the expression of CD163 and CD200R, two markers of M2-like macrophages (Figure 5c, d). By flow cytometry, we observed that STAT6 phosphorylation was significantly inhibited in SOCS3-overexpressed macrophages (Figure 5e). These results indicate that SOCS3 induces M1-like polarization but inhibits M2-like polarization in THP-1 macrophages. In SOCS3-overexpressed macrophages, *T. marneffei* infection led to significant upregulation of IL-10 and STAT6 phosphorylation, however, the up-regulation was inhibited compared with negative control macrophage (Figure 5b, e). In addition, we showed that *T. marneffei* infection significantly reduced the level of SOCS3 protein in SOCS3-overexpressed macrophages (figure 5f).Next, we measured the effect of SOCS3 on killing activity of macrophages. Compared with control cells, SOCS3-overexpressed THP-1 macrophages had stronger killing activity against *T. marneffei* (Figure 5g).

Taken together, we showed that SOCS3 plays an important role in the induction of M1-like polarization and in the inhibition of M2-like polarization in human THP-1 macrophages, but *T. marneffei* infection partially reversed these effects by the downregulation of SOCS3 expression.

### T. marneffei *infection induces SOCS3 protein degradation via tyrosine phosphorylation of SOCS3 protein.*

**Figure 6.**
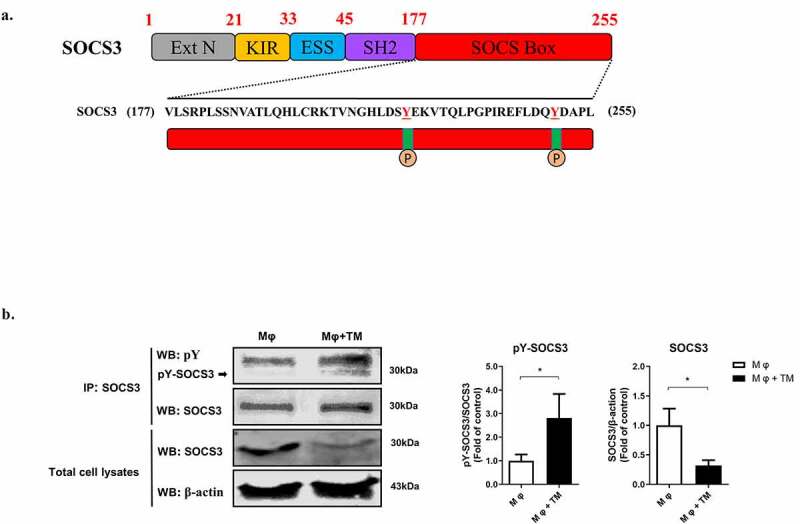
*T. marneffei* infection induces tyrosine phosphorylation of SOCS3 protein thereby enhancing SOCS3 protein degradation in THP-1 macrophages. The human THP-1 macrophages (PMA-treated) were infected with *T. marneffei* spores (MOI = 10) for 24 h. (a) The structure of SOCS3 protein and the amino acid sequence of SOCS BOX region. Tyrosine (Tyr204 and/or Tyr221) in the SOCS BOX region can be phosphorylated to initiate proteasome-mediated degradation of SOCS3 protein. (b) The effect of *T. marneffei* infection on tyrosine phosphorylation of SOCS3. The macrophages were lysised and were co-incubated with capture SOCS3 antibody for immunoprecipitation. Western blot was used to detect the levels of SOCS3 tyrosine phosphorylation and SOCS3 in *T. marneffei*-infected or uninfected macrophages. The relative pY-SOCS3/SOCS3 ratios were calculated and shown as fold of control. And the relative SOCS3/β-actin ratios were calculated and shown as fold of control (without *T. marneffei* infection). All data were showed as mean ± SD of results of three independent experiments (*, *p* < 0.05, **, *p* < 0.01, by Student’s t-test)

While the effect of *T. marneffei* on SOCS3 mRNA expression was variable (increased at beginning, then decreased) at different time points (Figure 4a), the levels of SOCS3 protein in *T. marneffei*-infected macrophages were consistently lower than control cells at all three time points. This suggests that the regulation of SOCS3 expression by *T. marneffei* infection mainly occurs at the protein level, specifically, via protein degradation. Previous studies have shown that phosphorylation of tyrosine (Tyr204 and/or Tyr221) in the SOCS BOX region of SOCS3 promotes proteasome-mediated degradation of SOCS3 protein [30] (Figure 6a). We assumed that *T. marneffei* infection causes SOCS3 protein degradation by inducing tyrosine phosphorylation of SOCS3 protein. To test this hypothesis, we used immunoprecipitation and western blot to measure the level of tyrosine phosphorylation of SOCS3 after infecting THP-1 macrophages with *T. marneffei* spores. We showed that SOCS3 tyrosine phosphorylation was significantly increased at 24 h post-infection, accompanied by significantly decreased SOCS3 expression in the infected cells (Figure 6b). This indicates that tyrosine phosphorylation of SOCS3 may be an important cause of *T. marneffei*-induced degradation of SOCS3 protein.

### *The TLR9 pathway is involved in* T. marneffei*-induced M2-like polarization of THP-1 macrophages.*

**Figure 7.**
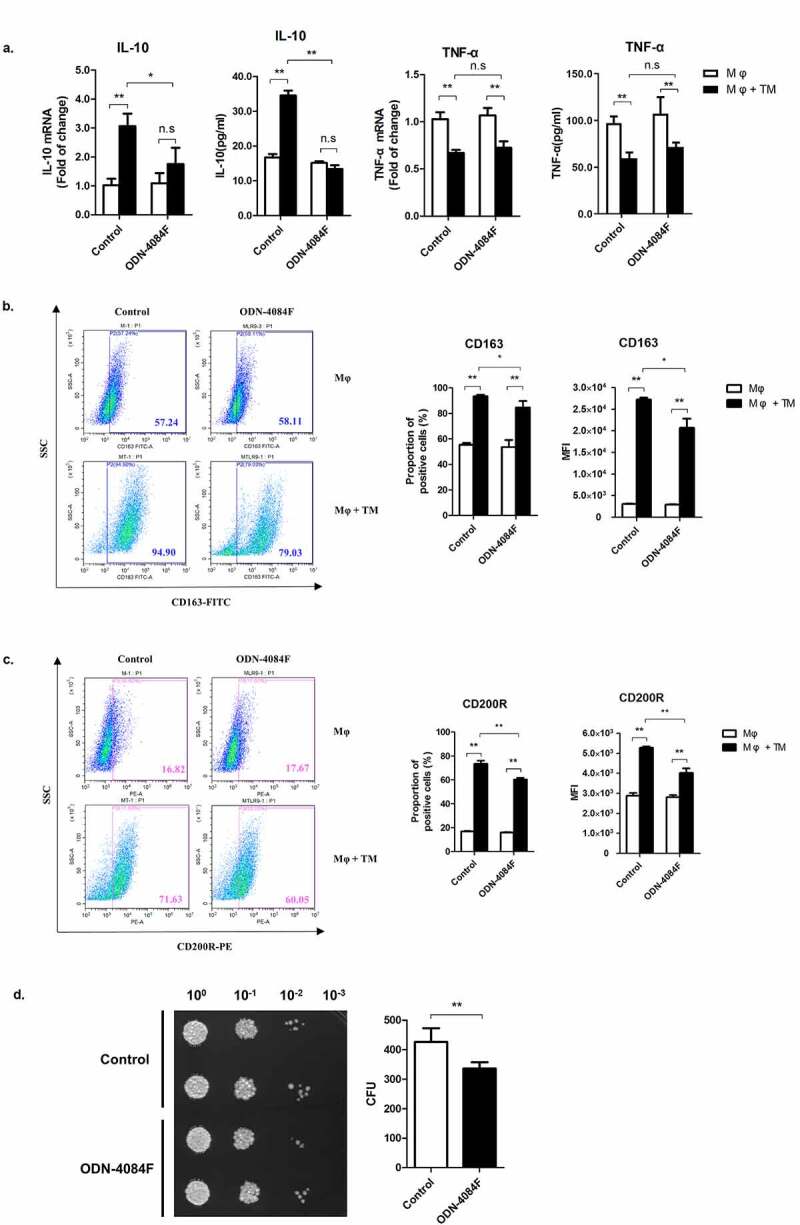
*T. marneffei* infection mediates M2 polarization of THP-1 macrophages by the TLR9 pathway. THP-1 macrophages were treated with 1 μM of ODN-4084 F or control ODN for 24 hr, then infected or uninfected with *T. marneffei* spores (MOI = 10) for 24 h. (a) Levels of TNF-α and IL-10 as measured by qRT-PCR and CBA. All the levels of mRNA expression were normalized to GAPDH and compared with respective timepoint controls. (b, c) Expression of CD163 (b) and CD200R (c) as measured by flow cytometry. (d) The effect of TLR9 on antifungal function of macrophages. Culture supernatants and cell lysates were collected, harvest *T. marneffei* by centrifugation. And the CFUs microdilution spot assay were conducted to detect the killing ability of macrophages to *T. marneffei*. All data were showed as mean ± SD of results of three independent experiments (*, *p* < 0.05, **, *p* < 0.01, n.s, no significant difference, by Student’s t-test)

Previous studies have shown that SOCS3 plays an important role in the regulation of M1-like polarization [19]. However, the fact that *T. marneffei* reduces SOCS3 expression does not mean that SOCS3 degradation is sufficient for the induction of M2-like polarization. Other factors may contribute to *T. marneffei*-induced M2-like polarization. The TLR9 pathway has been reported to be involved in M2-like polarization of macrophages, and TLR9 is a key receptor that recognizes *T. marneffei* infection. We hypothesized that TLR9 is involved in *T. marneffei*-induced macrophage M2-like polarization. To test this hypothesis, we suppressed TLR9 receptor of THP-1 macrophages via its antagonist, ODN-4084 F, and then infected the cells with *T. marneffei* and measured the production of TNF-α and IL-10. We showed that the suppression of the TLR9 pathway completely blocked IL-10 upregulation in response to *T. marneffei* infection. However, it insignificantly increased the expression of TNF-α (Figure 7a). The suppression of the TLR9 pathway partially inhibited *T. marneffei*-induced upregulation of CD163 and CD200R (Figure 7b, c). Importantly, the role of the TLR9 pathway in *T. marneffei*-induced M2-like polarization was evidenced by the fact that TLR9 antagonist improved the killing activity of THP-1 macrophages against *T. marneffei* (Figure 7d). These data showed that TLR9 mediates M2-like polarization of THP-1 macrophages in response to *T. marneffei* infection.

### T. marneffei*-induced human THP-1 macrophages M2-like polarization is additively regulated by SOCS3 and the TLR9 pathway.*

**Figure 8.**
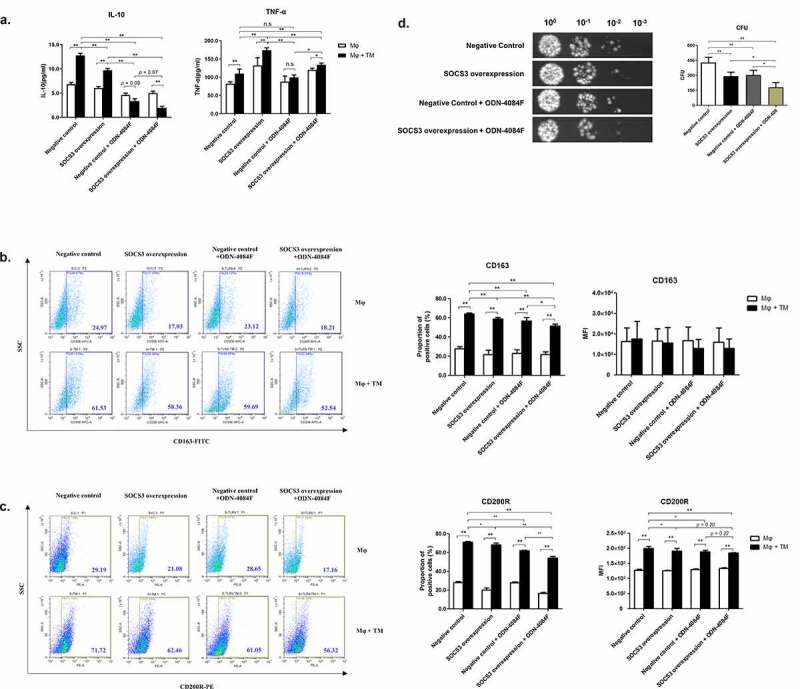
TLR9 and SOCS3 additively mediate M2-like polarization in *T. marneffei*-induced THP-1 macrophages. SOCS3-overexpressed macrophages or control cells were treated with 1 μM of ODN-4084 F or control ODN for 24 h, then infected or uninfected with *T. marneffei* spores (MOI = 10) for 24 h. (a) Levels of TNF-α and IL-10 were detected by CBA. (b, c) Expression of CD163 (b) and CD200R (c) are measured by flow cytometry. (d) The effect of TLR9 on antifungal function of SOCS3 overexpression macrophages and control cells. Culture supernatants and cell lysates were collected, harvest *T. marneffei* by centrifugation. And the CFUs microdilution spot assay were conducted to detect the killing ability of macrophages to *T. marneffei*. All data were showed as mean ± SD of results of three independent experiments (*, *p* < 0.05, **, *p* < 0.01, n.s, no significant difference, by Student’s t-test)

We further explored the relationship between SOCS3 and TLR9 in macrophage M2-like polarization. First, we suppressed the TLR9 pathway in SOCS3-overexpressed THP-1 macrophages, then infected the cells with *T. marneffei* spores and measured the production of TNF-α and IL-10. Suppression of the TLR9 pathway or/and SOCS3 overexpression significantly reduced the production of IL-10 in response to *T. marneffei* infection, and partially blocked the upregulation of TNF-α (Figure 8a). In addition, TLR9 suppression or/and SOCS3 overexpression partially inhibited *T. marneffei*-mediated upregulation of CD163 and CD200R (Figure 8b, c). Similarly, both TLR9 suppression and SOCS3 overexpression enhanced the killing activity of macrophages against *T. marneffei*, respectively (Figure 8d). Moreover, TLR9 suppression in SOCS3-overexpressed macrophages showed the strongest ability to kill *T. marneffei* (Figure 8d), indicating that the weakening of M2-like polarization is not conducive to the survival of TM. Furthermore, the results above are also mutually verified with the related results of Figure 5 and Figure 7. Collectively, we demonstrated that *T. marneffei*-induced M2-like polarization of human THP-1 macrophages is additively regulated by SOCS3 and the TLR9 pathway.

## Discussion

In this study, we demonstrated that *T. marneffei* resisted the direct fungicidal effects of macrophage by inducing macrophage M2-like polarization. We showed that SOCS3-STAT6 and the TLR9 pathways directly participated in this process. Specifically, *T. marneffei* infection downregulated the level of SOCS3 protein, thereby disinhibiting the effect of SOCS3 on STAT6 phosphorylation and facilitating macrophage M2-like polarization. We observed that SOCS3 protein is actually a positive regulator of the M1-like polarization in human macrophages. Moreover, M2-like polarization of macrophages induced by *T. marneffei* infection is also mediated by TLR9 activation.

Macrophage polarization has emerged as a key role in antifungal activity of macrophages. Similar to other fungal and intracellular pathogens, we observed the ability of *T. marneffei* to shift human macrophage polarization status from M1-like to M2-like to evade the antifungal activity of THP-1 macrophages. The intracellular environment of macrophages is harsh by design, including oxidative stress, heat stress, and nutrient deprivation [31,32]. *T. marneffei* has been shown to develop strategies to withstand these stresses. These include challenging oxidative stress via nuclear localization of transcription factors and signal transduction via phosphorylation, responding to heat stress by initiating yeast morphogenesis, and using the glyoxylate cycle to overcome the dilemma of nutritional deprivation [33,34]. Inducing M2-like polarization of macrophage is another effective strategy for *T. marneffei* to resist antifungal killing of macrophages, rendering them permissible to fungal persistence and dissemination. Of note, *T. marneffei*-induced M2-like polarization was not only observed in human macrophage cell line (THP-1), but also in human monocytes/macrophages (Figure 2), partially implying that *T. marneffei*-induced macrophage M2-like polarization may be a common phenomenon in human macrophages. In addition, our results suggested that there appears to be a population of CD163-low macrophages arising in *T. marneffei*-induced group at 48 h (Figure 2b), which was similar to the results of other studies [35,36]. We speculate that macrophages may have a negative feedback regulation mechanism, and there may be an upper limit to the degree of M2-like polarization of macrophages.

In fact, it must be admitted that the black/white functional phenotypes, M1/M2 dichotomy, only represent the two extremes of differentiation states. The macrophage polarization is very complex, involving internal, external, and tissue environmental stimuli, including cytokines, growth factors, fatty acids, prostaglandins, and pathogen-derived molecules. Human M1-like and M2-like macrophages can switch between each other once the micro-environmental conditions change, therefore, the separation between human M1-like and M2-like macrophages is actually continuous, which means the boundary is not clear. In the process of *T. marneffei*-host macrophages interaction, IFN-γ is a crucial factor for macrophages to defense the pathogen. Previous studies in murine macrophages showed that IFN-γ could up-regulate M1-like factors even infected with *T. marneffei* [37], which are consistent with our results of human M1-like macrophage (PMA/LPS/IFN-γ-treated) infection model. Thus, IFN-γ has significant therapeutic potential to inhibit *T. marneffei* infection. Notably, our research found that *T. marneffei* has the potential to affect M1-like to M2-like repolarization, which is an effective survival strategy.

Our finding that SOCS3 shifts macrophage polarization from M2-like to M1-like phenotype is similar to a previous study [17]. Using adoptive transfer of SOCS3-silenced macrophages in a mouse peritonitis model, the investigators demonstrated that SOCS3 drives the production of M1-induced cytokines (TNF-α, IL-1β), while reducing the expression of M2-induced IL-10 [19]. Our finding is also consistent with another study in rat bone marrow-derived macrophages, in which silencing of SOCS3 led to down-regulation of the M1-like polarization gene [38]. Interestingly, there are species-specific and cells-specific differences in the role of SOCS3 in driving macrophage polarization [18]. Specifically, most studies showed that SOCS3 is a negative regulator of M1-like polarization in bone-marrow-derived macrophages from mouse and glioma patients, but few studies showed that it is a positive regulator of M1-like polarization. in human THP-1 macrophages and rat bone marrow-derived macrophages [39–42]. The reason for the opposite result was not clear, which the possible reason as follows, firstly, the previous researches used different experimental methods for the inhibition expression of SOCS3 in macrophages. The completely deletion of SOCS3 in macrophages may cause compensatory alterations in other signaling pathways or proteins, which are not observed using siRNA to knocked down the function of SOCS3. Secondly, the macrophages of different species and tissue are respond differently to stimulus factors. Overall, a unifying role of SOCS3 is macrophage polarization across species has not been established, and further research are need to better define the role and mechanism of SOCS3 in the regulation of macrophage polarization against fungal infections.

As an important member of SOCS protein family, SOCS3 is a cytokine-inducible negative regulator of cytokine signaling pathways. Since the IL4/IL13-driven the JAK-STAT6 pathway directly promotes macrophage M2-like polarization [17,18], we hypothesized that the JAK-STAT6 pathway is involved in *T. marneffei*-induced M2 polarization of macrophages. Our results confirmed this hypothesis showing that *T. marneffei* infection activated the JAK-STAT6 pathway by downregulating the level of SOCS3. Our results further found that tyrosine phosphorylation of SOCS3 could be selectively activated by *T. marneffei* infection, and tyrosine phosphorylation within the SOCS3 box region directly induced SOCS3 degradation [30,43,44]. Our data therefore provided a complete chain of evidence that *T. marneffe*i infection degrades SOCS3 protein, thereby disinhibits the effect of SOCS3 on STAT6 phosphorylation, and facilitates M2-like polarization of macrophages which is beneficial for the survival of *T. marneffei* in THP-1 macrophages.

Besides the JAK-STAT6 pathway, TLR9 has been shown to be involved in M2 polarization [26]. Traditionally, TLR9 receptor mediates cellular response to cytidine phosphate guanosine (CpG) in fungal DNA to trigger an innate immune response [45–47]. Interestingly, recent research has shown that in addition to DNA, TLR9 directly recognizes chitin of fungal cell wall, thereby increases the production of IL-10 [16,48]. In the present study, the inhibition of the TLR9 pathway by ODN-4084 F significantly blocked the upregulation of IL-10 and enhanced phagocytosis and killing activity of human THP-1 macrophages upon *T. marneffei* infection. The down regulation of CD163 and CD200R by ODN-4084 F further support our insertion that the TLR9 pathway is important for the regulation of M2-like polarization in THP-1 macrophage. However, the specific molecular mechanism by which *T. marneffei* activates the TLR9 pathway is still unclear. We can only speculate the fungal DNA or/and chitin on *T. marneffei* may be involved in the activation.

There are several limitations in the present study. First, only one cell line, THP-1 macrophage, was used. Considering the species-specific and cell-specific characteristics of SOCS3, it is unclear whether this mechanism is reproducible in other macrophage cell lines. Second, the SOCS3 knockdown cell line needs to be further studied the role of SOCS3 in macrophage polarization. Third, our in vitro experiments do not simulate the immune microenvironment in vivo. In vivo infection, the body’s immune cells, such as CD4^+^T cells, neutrophils, NK cells, and macrophages interact with each other to coordinate an antifungal response. Therefore, the question whether *T. marneffei* can evade immune killing by inducing macrophage M2-like polarization requires further verification in in vivo experiments.

Collectively, our data lead us to propose that *T. marneffei* avoids macrophage killing by modulating the SOCS3-STAT6 and the TLR9 pathways to induce M2-like polarization in human THP-1 macrophages. Our study reveals a novel mechanism by which *T. marneffei* evades the innate immune response, which may provide a therapeutic target for inhibition of infection and dissemination in *T. marneffei* infection.

## Materials and methods

### Cell line and peripheral blood mononuclear cells (PBMCs)

The human monocytic cell line THP-1 was purchased from Chinese Academy of Sciences Cell Bank. The THP-1 monocytes were stimulated with 50 ng/ml PMA for 72 hours for monocytes differentiation to macrophages, as previously described [49]. For M1 activation, macrophages were activated with LPS (100 ng/mL) and IFN-γ (20 ng/mL) for 24 h. For M2-like activation, macrophages were activated with IL-4 (20 ng/mL) for 24 h. A SOCS3-overexpressed THP-1 cell line was generated using lentivirus construction strategy. The lentivirus vector containing green fluorescent protein (GFP)-SOCS3 sequences was constructed by Genechem Company (Shanghai, China). Peripheral blood mononuclear cells (PBMCs) were isolated from healthy volunteers. Briefly, blood sample was collected from healthy subjects (n = 3). PBMCs were isolated by Ficoll-Paque Plus (GE Healthcare) density centrifugation according to the manufacturer’s instructions. The study, including the recruitment of healthy volunteers, was approved by the Institutional Review Board and Human Ethics Committee of Guangxi Medical University, and the informed consents were obtained from all the subjects. THP-1 cells and PBMCs were cultured in RPMI 1640 medium containing 10% FBS, 100 U/mL penicillin, and 100 μg/mL streptomycin. THP-1 differentiated macrophage was maintained in 10% heat-inactivated FBS in DMEM with 100 U/mL penicillin and 100 μg/mL streptomycin. The cells were cultured at 37°C and 5% CO_2_. The medium was changed every 3 d.

### Fungal strain and media

Experiments were conducted using *T. marneffei* strain L0, which was separated from an HIV and *T. marneffei* co-infected patient. *T. marneffei* was identified by both standard culture morphology and by PCR-based sequence analysis of the 16S-23S rRNA internal transcribed spacer region (ITS). *T. marneffei* was plated onto *Potato dextrose agar* (PDA) agar at 27°C. The conidia were harvested by suspension in PBS after incubation for 7 to 10 d on PDA. The purified conidia were separated by filtering through sterile glass wool. The conidial suspension with the concentration of 10^7^ conidia/ml was prepared for subsequent experiments.

### T. marneffei *infection.*

The purified conidia suspended in RPMI 1640 or DMEM medium with 10% FBS were added to PBMCs or THP-1 macrophages, respectively (MOI = 10), as previously described [50].

### T. marneffei *survival assay.*

In brief, THP-1 macrophages were incubated with T. *marneffei* (MOI = 10) for 24 h at 37°C and 5% CO_2._ For ODN-4084-F related experiments, cells were seeded on 96-well plates at 1 × 10^5^ cells per well, rest of which were seeded on 24-well plates at 3 × 10^5^ cells per well, ensure that cells were seeded at 90% confluence into different plates [51,52]. The supernatant was collected, and the cells were lysed in sterile water to release the fungus. Four gradient serial dilutions (10°, 10^1^, 10^2^, and 10^3^) were performed and plated onto YPD agar. Numbers of fungal colony-forming units (CFUs) were counted after 24 h incubation at 30°C.

### Real-time reverse transcription PCR (qRT-PCR)

qRT-PCR was carried out as previously described [53]. In brief, total cellular RNA was extracted using Universal RNA Extraction Kits (Takara). Then the Reverse Transcription Kit (Takara) was used to reversely transcribe RNA to cDNA, according to the manufacturer’s instructions. Gene expression was examined with a StepOne Plus real-time PCR system (Life Technologies) with SYBR Green PCR Master Mix (Takara). The values were normalized to GAPDH expression.

### Western blot

Western blot was carried out as previously described [53]. Briefly, total cell lysates were prepared using radioimmune precipitation assay (RIPA) buffer (MultiSciences Biotech) with 1% protease inhibitor cocktail (Asvio Technology). The primary antibodies used in this study were listed as follows: rabbit-anti-β-actin (1:1000) (CST), rabbit-anti-SOCS3 (1:1000) (CST), mouse-anti-Phosphotyrosine (1:500) (Millipore). The secondary antibodies were horseradish peroxidase-conjugated goat-anti-rabbit IgG (1:15,000) (LI-COR Biosciences) or goat-anti-mouse IgG (1:15,000) (LI-COR Biosciences), respectively. The immunoreactive bands were visualized by Odyssey CLx Infrared Imaging System (LI-COR Biosciences). The densitometric analysis of blots was performed by Image Studio Ver 5.2 software (LI-COR Biosciences). The values were normalized to those of control β-actin.

### Flow cytometry

Flow Cytometry was carried out according to the manufacturer’s instructions (BD Biosciences). In brief, for cell surface staining, the cells were incubated with Human BD Fc Block^TM^ at room temperature for 10 min, then antibodies were added, and incubation maintained for 30 min in the absence of light at 4°C. For intracellular staining, the cells were fixed with incubating pre-warmed BD Cytofix^TM^ Fixation Biffer for 10 min at 37°C. Then the cells were incubated with BD Phosflow^TM^ Perm/Wash Buffer III for 30 min at room temperature. At last, antibodies were added, and incubation maintained for 60 min at 4°C protected from light. For Intracellular cytokine detection, cells were treated with 5 μM of Brefeldin A (BFA) for 6 h before the detection. For Cytometric Bead Assay (CBA), the cells were incubated with beads at room temperature for 1 h, then antibodies were added, and incubation maintained for 2 hr at room temperature protected from light. Fluorescence was detected with Beckman CytoFLEX FCM, and the data was analyzed by Beckman CytExpert 2.0 Software. The antibodies used in this study were listed as follows: BD Pharmingen^TM^ FITC Mouse Anti-Human CD163, BD Pharmingen^TM^ Alexa Fluor® 647 Mouse Anti-Human CD163, eBioscience^TM^ PE Mouse Anti-Human CD200R, and BD Phosflow^TM^ Alexa Fluor® 488 Mouse Anti-Stat6 (pY641). The CBA Flex Sets used in this study were BD^TM^ Rat TNF Flex Set and BD^TM^ Mouse IL-10 Flex Set. The SSC/FSC gate was used to excluded cell debris and then gating the interested cell population via specific antibodies. All gates in flow cytometry are defined by unstained control sample. The mean fluorescent intensity (MFI) was used for quantify the amount of target protein.

### Immunoprecipitation

Immunoprecipitation was conducted using PureProteome^TM^ Protein A/G Mix Magnetic Beads (Millipore). Briefly, the macrophages were lysed using lysis buffer (20 mM Tris-HCl pH 7.5, 150 mM NaCl, 1 mM Na2EDTA, 1% Triton, 1 mM EGTA, 1 mM NA_3_VO_4_) supplemented with 1% protease/phosphataes inhibitor cocktail (CST), and incubated on ice for 5 minutes. The supernatant was collected by centrifugation at 14,000 g for 10 minutes, and co-incubated with capture SOCS3 antibody (5 μg/10^7^ cells; Abcam) at 4°C overnight. The antibody-antigen complex was then added to the beads and incubated for 30 min at room temperature. Finally, adding the appropriate elution buffer for denaturing elution after washing the complex. The samples were heated to 95–100°C for 5 minutes, and then cooled on ice. Immunoprecipitated proteins were analyzed by western blot with a rabbit-anti-SOCS3 antibody (Abcam). To eliminate the interference of rabbit lgG heavy and light chains, Mouse Anti-rabbit lgG (Conformation Specific) (CST), which only reacts with native lgG and doesn’t bind to the denatured and reduced rabbit lgG heavy and light chains, were used as the secondary antibody for western blot.

### Statistical analysis

All experimental groups were performed in triplicates. Experimental groups were compared using Student’s *t* test for pair-wise comparisons and using one-way analysis variance (ANOVA) for multiple group comparisons. Data were shown as mean ± standard deviation (SD).

## Data Availability

The datasets generated for this study are available on request to the corresponding author.
